# Left-Sided Presentation of Acute Appendicitis in a Patient With Situs Inversus Totalis

**DOI:** 10.7759/cureus.40839

**Published:** 2023-06-22

**Authors:** Bassem Sous, Anisa Raidah, Faiz Syed, Nolberto Jaramillo, Toluwumi Olafisoye, Dean Olsen

**Affiliations:** 1 Emergency Medicine, Nassau University Medical Center, East Meadow, USA; 2 Surgery, New York Institute of Technology College of Osteopathic Medicine, Old Westbury, USA

**Keywords:** left lower quadrant pain, left-sided acute appendicitis, diagnosis of acute appendicitis, situs inversus totalis (sit), atypical appendicitis

## Abstract

Situs inversus totalis (SIT) is a rare condition characterized by mirror-image reversal of the thoracoabdominal organs. Although appendiceal pain is typically located in the right lower quadrant, in SIT, this can occur in the left lower quadrant. We present a case of a 40-year-old male with no medical history who complained of acute left lower quadrant pain, nausea, and vomiting with no other symptoms. A computed tomography scan revealed SIT and left-sided acute appendicitis (LSAA), which was managed surgically. This case highlights the importance of including appendicitis in the differential diagnosis for left lower quadrant pain.

## Introduction

Situs solitus is a term used to describe the normal anatomy, whereas situs inversus and situs ambiguous are used to describe complete reversal and abnormality of the left-right development, respectively [1]. First reported in the 1600s, ​​situs inversus totalis (SIT) is a condition in which there is a complete reversal of both the thoracic and abdominal organs [2]. It is rare, occurring in one per 5,000-10,000 live births and is inherited in an autosomal recessive fashion with incomplete penetrance. The cause is most commonly idiopathic; however, it can also be associated with primary ciliary dyskinesia (PCD), genetic factors, and other chromosomal changes. PCD is an autosomal recessive mutation in cilia, rendering them nonfunctional [3]. Kartageners is a type of PCD that can lead to symptomatic SIT with pulmonary and sinus disease since dynein protein mutations lead to ciliary dyskinesia and impaired mucociliary clearance [4]. SIT rarely affects the quality of life; thus, individuals with SIT are normally high functioning. However, patients afflicted with SIT have an increased risk of heart, hepatobiliary, and spleen malformations [5]. Laterality disturbances in these patients can also increase the risk of difficulties during procedures such as endoscopy [1]. The incidence of acute appendicitis in patients with SIT is between 0.016% and 0.024%. The differential diagnosis of acute left lower quadrant abdominal pain commonly includes diverticulitis, Meckel’s diverticulitis, renal colic, epididymitis, incarcerated or strangulated hernia, bowel obstruction, psoas abscess, cystitis, and ruptured ovarian cyst. Less commonly considered is acute appendicitis, one of the most common intra-abdominal surgical emergencies. This classically presents with right lower quadrant abdominal pain; however, in the setting of extremely rare intestinal malformations, such as SIT, the pain can present on the left side [6]. We present a rare case of left-sided acute appendicitis (LSAA) in a patient with SIT discovered on a CT scan.

## Case presentation

**Figure 1:**
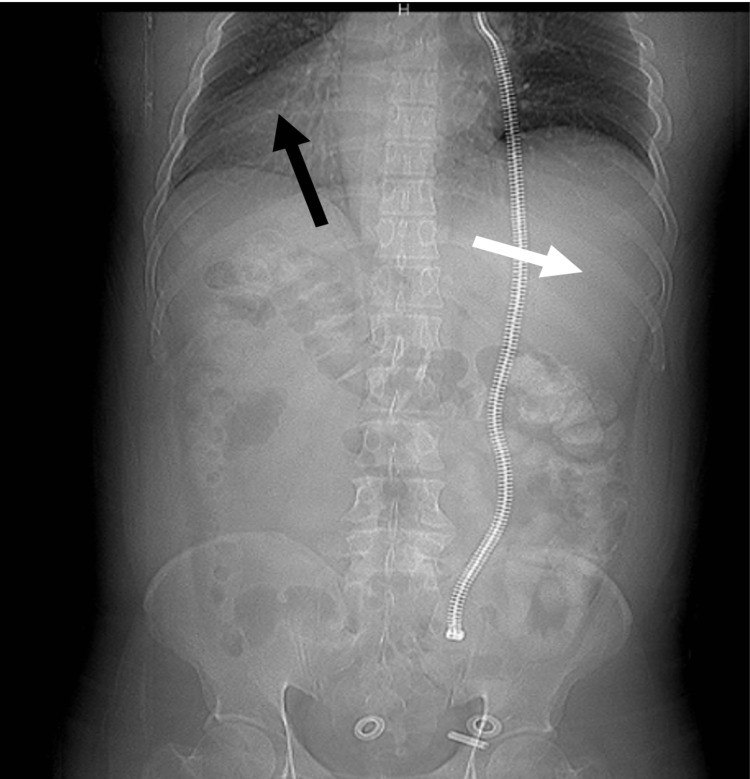
Chest radiograph showing situs inversus. A cardiac silhouette was observed in the right hemithorax (black arrow). A hepatic silhouette was observed in the left hemiabdomen (white arrow).

A 40-year-old male with no medical history presented to the emergency department complaining of intermittent, colicky, cramping left lower quadrant, and suprapubic pain for one day. He endorsed associated symptoms of nausea and non-bilious, non-bloody vomiting. He also drank eight beers and ate from a local restaurant the night prior with his wife; however, she was not ill. He denied any other symptoms, sick contacts, or recent travel. He denied taking any medications, surgical history, or family history. His vital signs were stable, and his physical exam was remarkable for moderate suprapubic and left lower quadrant tenderness. Laboratory studies revealed leukocytosis (14,700 cells/cubic millimeter, normal range: 4,500-11,000 cells/cubic millimeter) and neutrophilia (80.4%, normal range: 54-62% neutrophils). Chest radiograph revealed the cardiac silhouette present in the right hemithorax and hepatic silhouette observed in the left hemiabdomen, consistent with SIT (Figure 1).

**Figure 2:**
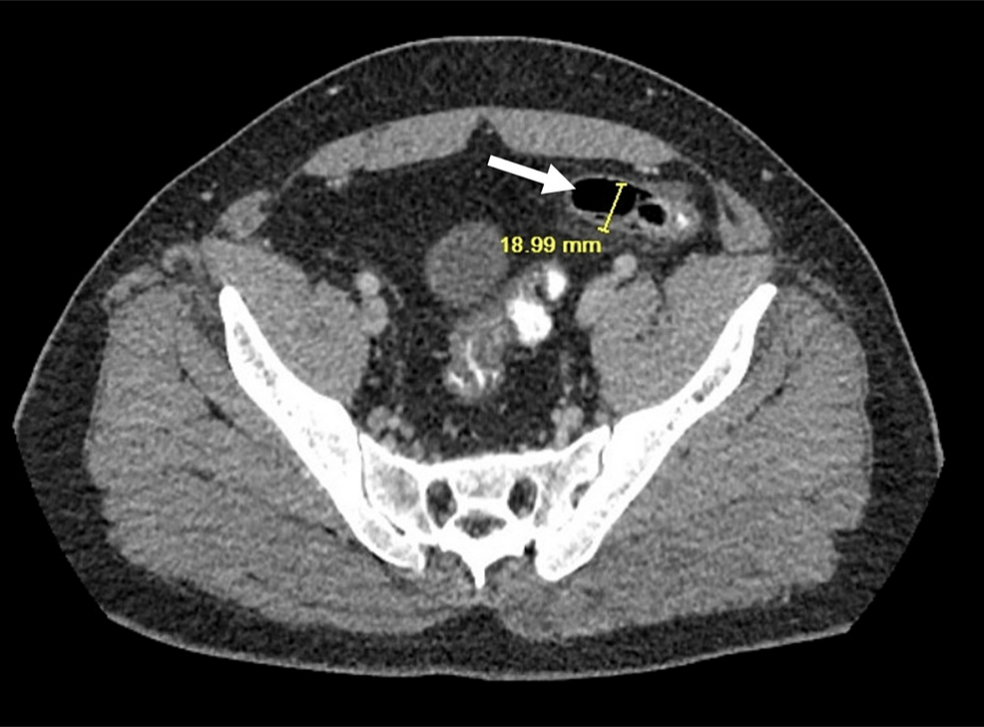
CT scan showing left-sided appendicitis (white arrow).

A CT scan of the abdomen and pelvis with contrast showed situs inversus with the aortic knob, cardiac shadow, and gastric air bubble all on the right and a dilated 1.9-cm appendix with surrounding mesenteric fat in the left lower quadrant of the abdomen (Figure 2).

**Figure 3:**
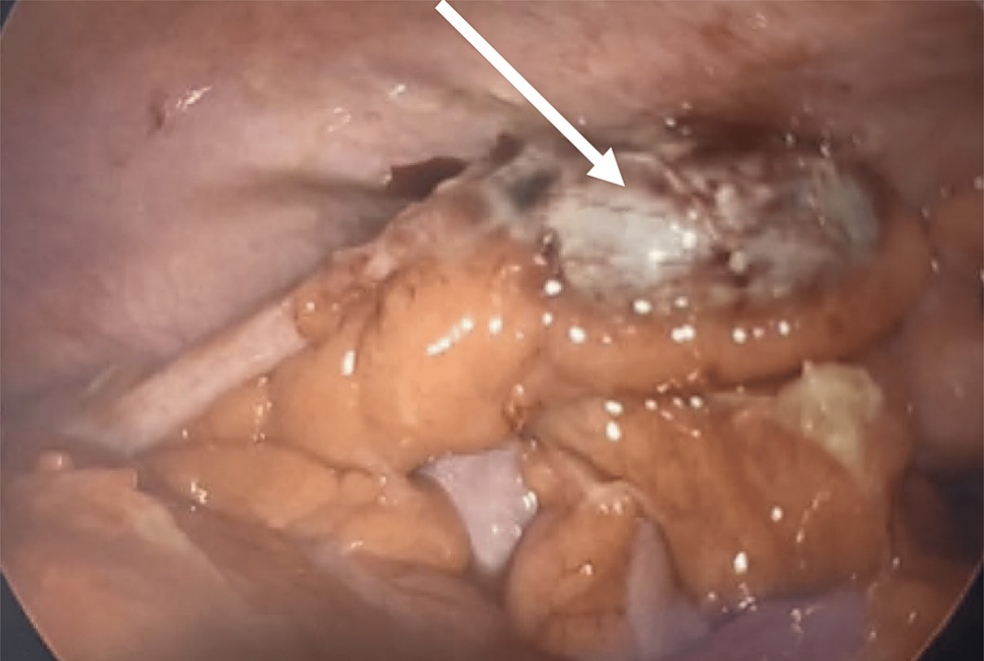
Laparoscopic image demonstrating left lower quadrant anatomy and appendix (white arrow).

The patient was subsequently admitted to the surgery floor for laparoscopic appendectomy and started on antibiotics and intravenous hydration. The surgery proceeded without complications, and a gross distally gangrenous appendix was removed (Figure 3).

Pathology results revealed necrotic appendicitis, and the patient was discharged from the hospital the following day with tramadol for pain control. One week later, he followed up in our clinic and was completely asymptomatic. His pain medication was discontinued, and he was encouraged to follow up as needed.

## Discussion

Approximately one-third of patients with appendicitis have an atypical pain location due to the various locations of the appendix, including retrocecal, subcecal, preileal, postileal, and pelvic. More rare locations include subhepatic, meso-celiac, mid-inguinal, and left-sided. LSAA is rare and associated with intestinal malformations, including SIT, midgut malrotation (MM), and cecal malrotation or a long right-sided appendix that projects into the left lower quadrant [6]. The majority of previous cases of LSAA were associated with a known history of SIT, left lower abdominal pain, and a diagnosis made preoperatively. In 18.4-31% of patients with SIT and MM, however, appendicitis led to right lower quadrant pain. Our patient was not aware that he had SIT, but did present with left lower quadrant pain and was diagnosed preoperatively. Surgical management for LSAA is done with laparoscopic removal of the appendix, which was performed in our patient [6]. A high index of suspicion must be maintained for this pathology, as acute appendicitis was not initially considered in our patient. The final diagnosis was made via a CT scan; therefore, the use of imaging is crucial in diagnosing LSAA. Clinicians must be careful to include LSAA in their differential diagnosis of acute left lower quadrant abdominal pain. 

## Conclusions

SIT is rare and must be considered when encountering reversed positioning of organs on diagnostic imaging. This case of LSAA in a patient with SIT highlights the importance of including it in the differential diagnosis of acute left lower quadrant abdominal pain. This discrepancy in location can lead to a delay in diagnosis of this common surgical emergency and increased morbidity associated with undiagnosed appendicitis; thus, physicians should be aware of this rare pathology.
